# Bridging Ecology and Microbiomes: Applying Ecological Theories in Host-associated Microbial Ecosystems

**DOI:** 10.1007/s40588-025-00246-z

**Published:** 2025-04-15

**Authors:** Clara Flores, Sophie Millard, Anna M. Seekatz

**Affiliations:** Department of Biological Sciences, Clemson University, Life Sciences Building 157 A, 190 Collings St, Clemson, SC 29634 USA

**Keywords:** Microbial ecology, Host microbiomes, Colonization, Neutral theory, Niche theory, Disturbance

## Abstract

**Purpose of Review:**

This review explores the application of classical ecological theory to host-associated microbiomes during initial colonization, maintenance, and recovery. We discuss unique challenges of applying these theories to host-associated microbiomes and host factors to consider going forward.

**Recent Findings:**

Recent studies applying community ecology principles to host microbiomes continue to demonstrate a role for both selective and stochastic processes in shaping host-associated microbiomes. However, ecological frameworks developed to describe dynamics during homeostasis do not necessarily apply during diseased or highly perturbed states, where large variations can potentially lead to alternate stable states.

**Summary:**

Despite providing valuable insights, the application of ecological theories to host-associated microbiomes has some unique challenges. The integration of host-specific factors, such as genotype or immune dynamics in ecological models or frameworks is crucial for understanding host microbiome assembly and stability, which could improve our ability to predict microbiome outcomes and improve host health.

## Introduction

Microbial ecosystems, or microbiomes, encompass bacteria, viruses, fungi, and other microscopic organisms that form complex multi-species communities within an ecosystem. Microbial ecosystems are broadly categorized as free-living (e.g., marine or soil) or host-associated (e.g., within insects, mammals, or plants), and significantly influence the health of their respective environment. For host-associated microbiomes, coevolution between microbes and their hosts has led to mutually beneficial symbiotic relationships, further adding complexity to microbial interactions. These interactions are shaped not only by microbe-microbe dynamics but also host-microbe interactions and host selective pressures.

Interest in host-associated microbiomes has surged in recent years, driven by their critical role in host biology and health and how to use this information to improve health outcomes [1]. Insights from community ecological frameworks, many of which were originally developed for macro-scale species interactions, have advanced our knowledge of microbial community dynamics and assembly [2]. However, a comprehensive understanding of the **deterministic** (e.g., environmental filtering, host immune pressures) and **stochastic** (e.g., random colonization, ecological drift) processes governing host microbiome assembly is essential for maintaining their stability, especially amid increasing human-driven disturbances. Traditional ecological theories must be adapted for microbial ecology, as microbes exhibit unique characteristics, such as higher genetic diversity, smaller size, rapid growth rates, and shorter evolutionary timescales, compared to macro-organisms. Defining whether macroecological rules are universal across microbiomes is thus valuable for improving their management, particularly in a rapidly changing world.

**Fig. 1:**
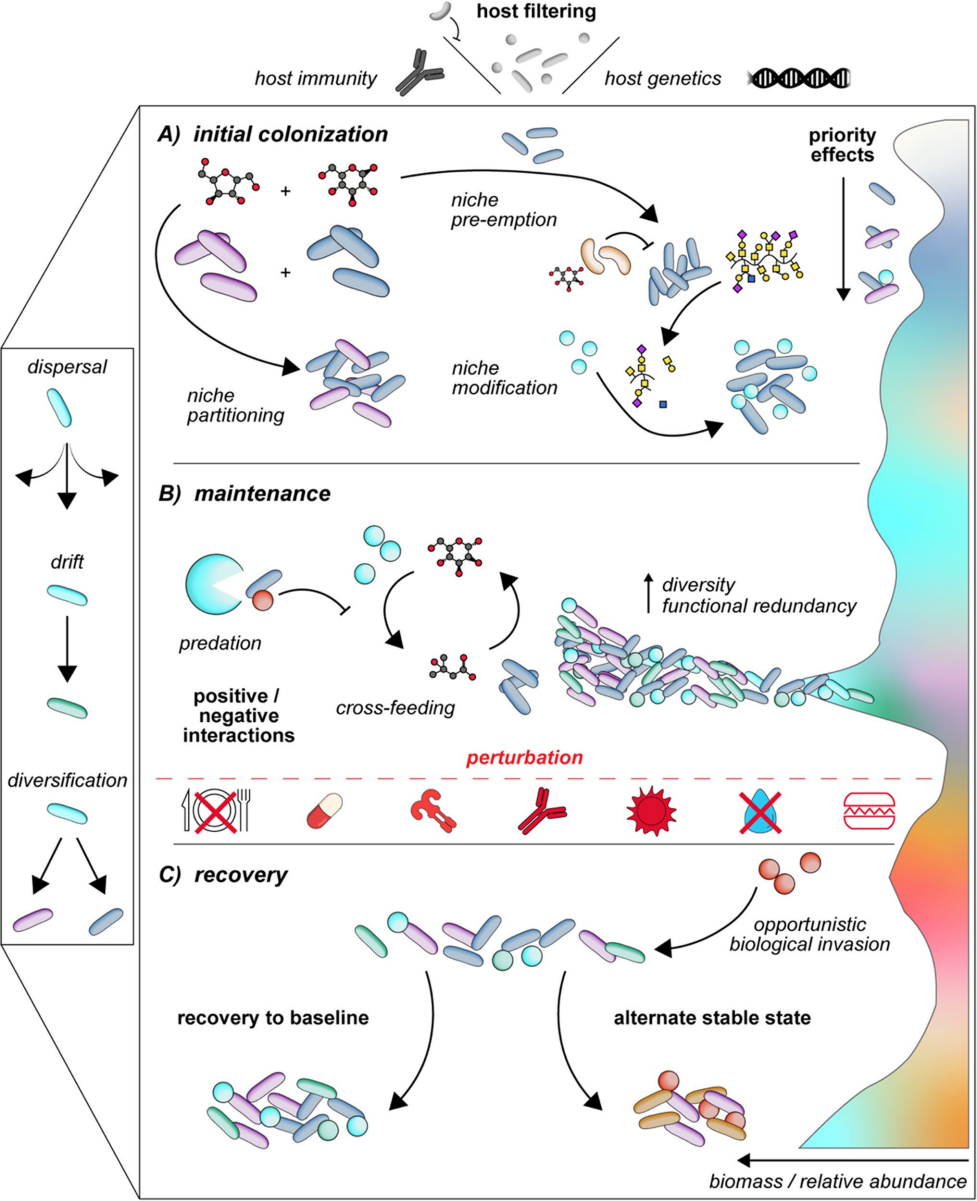
Ecological framework of colonization, maintenance, and recovery after perturbation in host-associated microbiomes. **A**) Host factors, such as immunity, genetics, or physiology, act as an initial barrier to colonizing microbes. Initial colonization is directed by the host niche, with early colonizers shaping succession of subsequent colonizers. **B**) As the environment is populated, additional ecological mechanisms contribute to maintenance of the population and persistence of species. This may include positive and negative interactions between species that influence the overall composition of the community, ideally populating the maximum carrying capacity with diverse organisms that can maintain homeostasis in the host. **C**) Recovery after perturbation will depend on the frequency, duration, and intensity of the perturbation. Large-scale disruptions may allow an open niche for opportunistic biological invasion or changes in community dynamics, with the community recovering to baseline or even alternate stable states. Throughout these dynamics, stochastic processes, such as dispersal, drift, and diversification, continue to shape the host microbiome

This review examines the application of classical community ecology theories in describing host-associated microbiomes, highlighting how these frameworks have been adapted for microbial community composition. We emphasize the stochastic and deterministic processes shaping microbial communities and discuss the challenges of their application to host-associated microbiomes during initial colonization, maintenance, and post-perturbation phases (Fig. 1). Finally, we outline host-specific factors that complicate the use of ecological theory in host-associated microbiomes.

## Initial Colonizers are Key Architects for Host-associated Microbiota

Most host-associated microbiomes are initially free of microbes, with both available resources and stochastic processes shaping selection. Early colonizers can exert a lasting influence on microbial community assembly, ultimately influencing host phenotype. For example, low-dose penicillin administered from birth in humans was shown to select for initial colonizers that affect ileal immune genes, predisposing individuals to obesity later in life [3]. In plants, microbial ethylene reduction by early colonizers was demonstrated to promote seedling growth, simultaneously decreasing stress tolerance [4]. In addition to initial selection, the sequence and timing of species arrivals contributes to early colonization dynamics and the resulting composition. Table 1 summarizes key terminology related to initial colonization and maintenance.

**Table 1:** Key terminology related to community assembly used in this review

Key theories	Definition	Reference
Deterministic processes	Directional forces that shape community structure and species interactions. These processes are driven by specific factors, such as natural selection, environmental conditions, species interactions, and specific traits which consistently influence community structure in a predictable manner	[5]
Dispersal	An ecological process in which one or more individuals move away from where they originated to a new location where they establish themselves and reproduce	[6]
Diversification	The process whereby a group of species within a community evolves to occupy distinct ecological niches, resulting in greater diversity of traits and functions within the community, often resulting from adaptation to different environmental conditions and resource availability	[6]
Ecological drift	The random fluctuations in species abundance within a community, driven by chance events such as births and deaths, leading to shifts in the relative frequencies of species without any particular competitive advantage or environmental selection pressure	[6]
Environmental selection	The deterministic result of local interactions between functionally different species and their environments	[6]
Host-filtering	A process where a host organism selectively influences the microbial organisms it interacts with, driven by various factors including, host traits, environmental factors, and transmission mode	[6]
Neutral theory	A concept that proposes that the relative abundance and composition of species within a community are primarily shaped by random processes, such as dispersal, drift, and diversification, rather than by deterministic factors like natural selection or competitive interactions	[7, 8]
Niche modification	A concept proposing that early-arriving species modify the types of niches that are available within a local site, which in turn influences the identities of late-arriving species that can colonize the community	[9]
Niche partitioning	The process where different species in a community divide up resources such as food, habitat, and time to minimize competition and allow for co-existence by utilizing different resources or occupying distinct parts of an ecosystem facilitating species diversity	[9]
Niche preemption	The concept in which early-arriving species diminish the availability of resources (nutrients, space, light, etc.), to other species, and in doing thereby restricting the local abundance that can be obtained by late-arriving species that need these resources to survive and reproduce	[9]
Priority effects	The influence that the order and timing of species immigration during community assembly can affect species abundances and interactions. The mechanisms of priority effects fall into two categories, niche preemption, and niche modification	[9]
Stochastic processes	Random events that influence the composition and dynamics of a community, often arising from unpredictable factors like random dispersal, birth, death, or environmental variations, leading to variation in species abundance and presence within a community	[5]

### Niche Partitioning And Resource Availability During Initial Colonization

The term ‘niche’, introduced by Joseph Grinnell in 1924, described an organism's potential to occupy a particular space and the behavioral adaptations required to do so [10]. Microbiome assembly in hosts, like any environment, is restricted to those adapted to the respective niche. Microbial niches are frequently defined by nutrient availability, a major selective driver of both initial colonization and long-term maintenance (described later). Aligning with the traditional ‘Grinnellian niche’, physical niches within the host also impose significant selective pressure on microbial colonizers. These niches vary widely, from leaf and root endospheres in plants, to specific anatomical sites, such as ceca or gut crypts in vertebrate species. As recently reviewed by Ludington, these niches are critical for the establishment of the gut microbiota [11]. For instance, in the human infant gut, initial colonization begins with aerotolerant bacteria such as *Enterobacteriaceae*, reflecting an aerobic environment, which subsequently shifts to dominance by anaerobic bacteria like *Bacteroidaceae* as the gut matures [12].

Host-specific processes further refine these physical niches through selective mechanisms collectively termed **host-filtering**, which include antimicrobial peptide production, hormonal signaling, and host physiology [13]. This type of selection has been observed across host species, from sea cucumbers [14] to vertebrates [15], whereby similar compositions arise in a host species due to their particular habitat. In some cases, hosts form specialized niches to support specific microbial symbionts, as observed in the bobtail squid’s light organ, uniquely adapted for colonization by the bioluminescent bacterium *Vibrio fisheri* [16]. Similarly, aphids house the bacteria *Buchnera aphidicola* within specialized cells termed bacteriocytes to provide essential amino acids lacking from their plant-sap diet [17], and legumes support nitrogen fixing bacteria called rhizobia in root nodules [18]. Related to host filtering, the concept of phylosymbiosis posits that a host’s microbial community more closely resembles that of its species than those of distantly related hosts, reflecting the co-evolution of microbiota alongside their hosts. Early studies on phylosymbiosis focused on *Nasonia,* a parasitoid wasp [19], and have been extensively reviewed elsewhere [20, 21].

### Priority Effects via Niche Preemption or Modification

During initial colonization, primary colonizers are critical in shaping the microbiota’s trajectory. Although many primary colonizers will be restricted by host-filtering, encounters with opportunistic microbes can also influence the trajectory of microbial composition. **Priority effects**, as described by Fukami, refer to the influence of early-arriving species on the establishment of subsequent colonizers [9]. Early colonizers can deplete shared resources, a process known as **niche preemption,** limiting the success of later-arriving species and potentially leading to competitive exclusion. Alternatively, they can modify the environment by creating or altering niches, enabling later-arriving species to exploit these newly available resources, a process known as **niche modification**.

The significance of priority effects on health has been demonstrated across various host systems. In healthy human infants, microbiome maturation follows a reproducible order, with specific genera appearing in a consistent order at defined stages of maturation [22]. Disruptions to this order have been implicated in several disease states, highlighting the importance of succession in initial colonization [23]. In the legume *Medicago lupulina,* the inoculation order of two *Ensifer* bacterial strains influenced both plant performance and rhizobia abundance in roots [24]. Priority effects also play a protective role by restricting colonization of unfavorable bacteria, as seen in neonatal chicks, where early colonizing *Enterobacteriaceae* effectively utilize available resources to outcompete pathogenic *Salmonella* [25].

Primary colonizers can also influence succession of community composition over time indirectly through host phenotype, such as host immune response and metabolism [26, 27]. For example, *Bifidobacterium longum* subspecies *infantis* produces indole- 3-lactic acid during early colonization, dampening host inflammatory responses in the infant gut [28]. Phenotypic changes in germ-free animals can be reversed through early-life colonization, underscoring the plasticity of host-microbiota interactions during critical developmental windows [29]. Studies comparing conventional and germ-free animals further demonstrate the profound effects of microbial colonization on host phenotype. Skin cells from germ-free mice exhibit altered expression of innate immune response genes [30], potentially explaining increased susceptibility to *Staphylococcus aureus* compared to their conventionally raised counterparts [31].

## Null and Niche Theories Explain Maintenance of Community Composition

Microbial community assembly continues to evolve dynamically following initial colonization. Even in relatively stable environments, interactions among microbial community members remain highly dynamic, necessitating ecological theories that account for both neutral processes and selection in established microbiomes. Processes shaping and maintaining community composition can be described by four core ecological processes—drift, dispersal, diversification, and selection. **Drift** refers to stochastic fluctuations in species abundances, **dispersal** involves the movement of organisms across spatial scales, **diversification** arises from both stochastic and deterministic mechanisms that increase genetic diversity potentially leading to speciation, and **environmental selection** reflects deterministic fitness differences among species [6]. Alongside these processes, positive and negative microbial interactions are critical for maintaining diversity in host-associated microbiomes [32].

### Null Theories and Stochastic Processes

Species abundances within microbial communities naturally fluctuate over time independent of selective pressures. Hubbell’s **neutral theory** posits that community composition is shaped by stochastic processes such as birth, death, colonization, and extinction, with speciation or immigration maintaining diversity within a community [7, 8]. Neutral theory assumes species equivalence in fitness and focuses on randomness as the primary driver of community composition. As such, these stochastic processes are central to neutral theory and its role in shaping microbial community structure, which can drive fluctuations in species abundance and contribute to both community divergence and individual genetic diversity.

Null models based on neutral theory have been applied to host-associated microbiomes with varying success. For example, studies on *Helicoverpa armigera* caterpillars revealed that neutral theory explained most bacterial taxa distribution patterns, though some taxa diverged from neutrality due to host selective pressures. *Enterococcus* species repeatedly appeared across caterpillar populations and were hypothesized to enhance host fitness by influencing immune response and potentially conferring insecticide resistance [33]. Similarly, Sieber et al. applied a neutral model across diverse host-associated microbiomes, finding that overall microbiota structure conformed to neutrality [34]. Jones et al. recently used probabilistic models in germ-free *Drosophila* inoculated with different bacterial combinations to conclude that stochasticity is an important part of community assembly [35].

Dispersal is a key process by which diversity accumulates in microbial communities. For instance, in healthy lungs, Venkataraman et al. found that dispersal of oral cavity microbes predominantly shaped the lung microbial community, aligning with predictions of a neutral model. However, this model was inapplicable in diseased states, where host selective pressures drastically altered composition [36]. Drift, or random species fluctuations, can also drive divergence. Comparisons of individual versus groups of the social spider *Stegodyphus* demonstrated that ecological drift drove microbiota divergence, whereby singly-contained spiders exhibited greater variability in microbiota composition and higher abundances of non-core symbionts [37]. With a synthetic microbial community, Fodelianakis et al. quantified ecological drift over time in vitro, revealing conditions of low dispersal and high selection that amplified stochastic effects on community composition [38].

Diversification, driven by stochastic and deterministic factors, introduces genetic variation and can lead to speciation when mutations and gene transfer occur across generations [39]. This process happens on a much shorter time scale for microorganisms, enabling them to rapidly acquire or lose functional traits. While experimental evolution demonstrates that bacterial speciation is likely [40], diversification in natural host-associated microbiomes can be difficult to study. Additionally, close interactions between host lineages and their associated microbial symbionts can lead to parallel diversification, also known as co-diversification. Moeller et al. explored various mammalian host lineages and found evidence supporting the co-diversification of microbial symbionts and both human and African ape hosts [41].

### Resource Availability and Niche Partitioning

As with initial colonization, the host-associated environment and selection continue to shape microbiota during maintenance. Almost six decades after Grinnell introduced the term niche, Rolf Freter proposed an adapted nutrient niche theory to specifically describe nutrient niches in the gut. Freter proposed that nutrient availability acts as a selective pressure, enabling species best equipped to exploit limiting resources to persist [42]. Recent research shows that nutrient niches within a host are highly specialized and can be subdivided, creating distinct microhabitats that support diverse communities. For instance, within the intestines, nitrate-utilizing microbes dominate epithelial cell-associated niches [43], while mucin-degrading species thrive in the outer mucus layer [44]. Resource competition initially favors colonizers best suited to exploit limiting resources but over time, it can drive community members to adapt and utilize alternative or unexploited niches [45]. Across host species, transitions from birth to weaning highlight how shifts in resource availability drive taxonomic and functional changes within microbiomes [46, 47].

### Integrating Microbial Interactions into Community Assembly

The maintenance of community composition, and by extension diversity, can also be explained by ecological processes that emphasize interactions between individual microbes. These interactions, ranging from positive (e.g. where a species promotes another species’ growth) to negative (e.g. where a species inhibits another species’ growth), play a crucial role in shaping the structure and relative composition within host-associated microbiomes, discussed in greater detail in another review of the mammalian gut microbiome [48].

Many interactions within host-associated microbiomes are classified as symbiosis, or the interaction between organisms living in close proximity. One positive interaction includes cross-feeding, where metabolites produced by one organism may be used by another, building a connected, more diverse community that may be better equipped to resist invasion and external perturbations. The ecological implications of cross-feeding within gut microbial communities are thoroughly reviewed by Culp and Goodman [49]. While symbiosis often benefits both partners, it also encompasses interactions where only one organism benefits (commensalism) or where one is harmed (parasitism).

Negative interactions, including competition and predation, also influence diversity and shape community assembly [48]. Predation has been well-studied in animal populations and is recognized as a key factor shaping animal communities. In microbiomes, instances of predation have been observed [50, 51], but its broad impact on community structure within a host remains less understood. Lotka-Volterra competitive models, initially developed to describe predator–prey dynamics [52], integrate population-level dynamics into microbial community assembly. The generalized Lotka-Volterra (gLV) framework, extends traditional competitive models by incorporating both positive and negative interactions [53] and is often used to study dynamics and drivers of complex microbial communities [54, 55].

## Host and Microbial Factors Dictate Community Assembly after Disturbance

Host-associated microbiomes frequently incur large-scale disruptions such as antibiotic treatment, dietary shifts, infections, and environmental stressors, which drastically alter community structure and function. Key terminology related to reassembly after these disruptions is summarized in Table 2. Optimally, ecological models could be used to predict outcomes following microbiome disruption to improve its recovery or beneficial trajectory. Yet, disturbances introduce complexities that challenge the predictive potential of models originally developed to describe stable states in healthy hosts. Furthermore, specific characteristics of disturbances—such as their frequency, duration, and intensity—play a variable influence on community trajectories and determine whether stability is maintained, disrupted, or reestablished in a novel configuration.

**Table 2:** Key terminology used when examining community response to disruption

Theory/Model	Definition	Reference
Functional redundancy	The concept that multiple species within a community can perform the same or similar function	[56]
Ecosystem resilience	The ability of a biological community to withstand, adapt to, and recover from environmental disturbances or changes, maintaining its structure and function even after experiencing a disruption	[57]
Intermediate disturbance hypothesis	Suggests that local species diversity is maximized when ecological disturbance is neither too rare nor too frequent	[58, 59]
Biological invasion theory	The study of how a non-native species, introduced to a new ecosystem, establishes itself and rapidly expands its population, potentially disrupting the existing community structure	[60]

### Ecosystem Resilience and Functional Redundancy in Recovery

Ecological theory suggests that species must differ in traits or relative environmental fitness; otherwise, competitive exclusion would eliminate less fit species. However, microbial communities often exhibit **functional redundancy**, with multiple species performing similar roles which maintains ecosystem function even when composition changes after perturbation [56]. Despite the benefits of having alternate, or redundant, functions to replace those lost after disturbances, the degree to which functional redundancy buffers against disruptions depends on the nature and scale of the disturbance. For example, transient shifts in species composition may preserve function, whereas more severe or prolonged disturbances can overwhelm this buffer and destabilize the community [57].

In host-associated microbiomes, short-term or infrequent disturbances often produce reversible shifts in community composition while maintaining functional capacity and host health. For example, dietary interventions can temporarily alter microbial composition, but typically revert to baseline once the intervention ends, provided the disturbance does not significantly reduce community diversity [61]. Similarly, specific taxa might transiently expand or decline in response to environmental disruptions, but functional stability is preserved as a result of functional redundancy. The **intermediate disturbance hypothesis** (IDH) offers a framework to understand how disturbances influence community structure. IDH suggests that maximal biodiversity occurs with frequent, moderate disturbances [58, 59]. While this has been demonstrated in environmental microbiomes [62, 63], studies in host-associated microbiomes are limited. Notably, Romer et al. found evidence of IDH in snake microbiomes, where diversity and richness increased following pathogen introduction [64], while Shao et al. observed increased microbiome diversity in Chinese mitten crabs in response to salinity changes, further supporting IDH [65].

### Opportunistic Invaders

Disturbances can also create ecological openings for opportunistic invaders to colonize. For example, antibiotic treatment often reduces microbial diversity in the gut, leaving niches open for pathogenic species to exploit and outcompete native microbes [66]. In amphibians, drought-induced microbiome disruptions were linked to increased pathogen susceptibility [67], and pollution-driven simplification of the red snapper microbiome was found to make the community more vulnerable to invaders [68]. These patterns align with **biological invasion theory** [60], which provides a framework for understanding how disturbances mediate the success of invaders in host-associated microbial ecosystems. In this context, the success of invading microorganisms, whether pathogenic or beneficial, depends on multiple factors including environmental conditions, nutrient availability, and resident community structure [69]. Reduced competition and available niches following disturbances facilitate invader establishment while high diversity and functional redundancy can act as barriers to invasion by promoting **ecosystem resilience**.

### Recovery and Secondary Succession

Recovery after disturbance involves secondary succession, where pre-existing elements impact community structure, as opposed to primary colonization of a naive environment. As with initial colonization, priority effects heavily influence the trajectory of community regrowth [70]. After disruption of the gut microbiome, keystone species play a critical role in the early stages of recovery by modifying the nutritional environment to support later colonizers [71]. For example, Weiss et al. demonstrated in a mouse model that keystone species within a synthetic gut community not only facilitated recovery but also modulated the host response, indirectly influencing the trajectory of community assembly [72]. Similarly, short-term antibiotic treatment in another murine model was shown to induce long lasting effects on metabolic interactions, underscoring the lasting influence of early microbial shifts on community dynamics [73]. In a study comparing microbiome recovery across environments, Jurburg et al. found that environmental microbiomes were more likely to enter an alternative state compared to original baseline in mammalian-associated microbiomes, suggesting that host-imposed filtering can overcome stochastic effects such as drift or dispersal [74]. Nonetheless, response to certain perturbations, such as antibiotic treatment in humans and other mammals, is highly individualized, indicating that the host ultimately drives variation and potential alternate states during recovery from larger disruptions [75].

## Challenges in Ecological Theory of Host-associated Microbiomes

Host-associated microbial ecosystems exhibit unique characteristics that challenge the broad application of ecological models to all microbiomes. For instance, ecological models in host-associated microbiomes must also consider host-specific factors such as genotype and immune responses (including both innate and adaptive), which on their own are highly individualized and dynamic over time. Advances in sequencing-based techniques, simplified experimental models, and predictive modeling frameworks are continually improving the application of ecological theory to complex systems. Despite these innovations, significant challenges remain in creating models that are both accurate and generalizable across different host systems and beyond.

### Technological

#### Sequencing-based Approaches

Advances in next-generation sequencing (NGS) have expanded our ability to characterize microbiome dynamics, including nucleotide-level resolution to track strain variation and adaptation, beyond cultivable organisms [76]. Despite these benefits, NGS still has inherent limitations that complicate its application in microbial ecology, outlined in detail by Prosser [77]. Even for NGS approaches that supply ‘functional’ information (i.e., metagenomics, metatranscriptomics), accurate modeling can only be conducted on annotated genes, which are still underrepresented in databases. Many modeling frameworks also require absolute abundance data, which NGS methods frequently fail to capture accurately. Efforts to overcome these limitations include incorporating calculations that estimate biomass into modeling parameters to enhance predictive accuracy [78]. Additionally, advances in machine learning approaches can enable integration of complex variables, such as longitudinal data, into microbiome models. Medina et al. reviewed machine learning applications in microbiome research, emphasizing its potential to uncover patterns and interactions that traditional models may overlook [79].

#### Simplified Experimental Systems

Organisms with less diverse or simplified natural communities continue to be valuable for studying microbiota assembly in a host. Indeed, many of the interpretations discussed above have relied on organisms with simpler microbiomes, from *C. elegans* models in the lab [80] to sea cucumbers in nature [14]. The use of defined bacterial consortia, either in in vitro or germ-free host systems, provide controlled environments to dissect ecological processes. These approaches have been used to validate ecological theories in plants [4], flies [35], and mice [72], as well as extrapolate ecological data to their natural or more complex microbiome [81]. While these systems have proven invaluable for understanding processes that govern microbiota assembly, simplified models may not fully capture the complexity observed in natural microbial ecosystems. Furthermore, germ-free and model approaches rely on available cultivars and a matched host environment. For instance, conclusions derived from mice with humanized microbiota may not realistically recapitulate human microbiomes [82]. Efforts to improve models include improving cultivation of target organisms [83] or the ability to genetically manipulate model hosts to better resemble their host counterpart [84]. Increased improvements in sequencing-based techniques and genetic manipulation, such as using CRISPR-Cas, are expected to improve generalizability of results from simplified to complex systems.

#### Predictive Modeling

Within a host, continuous metabolic exchange between the host and microbes can complicate predictions of functional interactions and accurate measurement of end-products. However, understanding this exchange, along with the intermediate products generated during these reactions, is crucial for characterizing the full scope of interactions. For example, Kurkijan et al. demonstrated through in silico experiments that accurately modeling invasion success requires considering not only species abundance data but also levels of their interaction-mediating chemicals [85]. Recent advancements in metabolic modeling address some of these constraints by integrating potential functional capabilities of microbes with ecological theories. Metabolic mapping has helped delineate functional niches, improving composition predictions for complex communities [86]. Brunner and Chia integrated metabolic models with gLV frameworks to study probiotic colonization, revealing the influence of metabolic interactions on strain establishment [87]. Similarly, Lambert et al. used metabolic modeling to improve classification of community interactions as competitive, neutral, or mutualistic [88].

### Biological

#### Host Genotype and the Immune Response

Perhaps the most distinctive features of host-associated microbiomes, compared to their environmental counterparts, is the direct influence of the host on the selection of microbes capable of colonizing or persisting in its environment. Host genotype, with its own variability across host species, is a non-specific means of host-filtering for certain microbes based on the host’s physiological attributes. Several studies demonstrate that host genotype influences microbiota composition across plants, invertebrates, and vertebrates, with variability of its effect observed across distinct parts of the host. In plants, rhizosphere microbiota has been observed to be shaped more by nutrient levels, whereas leaf-associated microbiota was more influenced by host genotype [89]. Host genotype has also been observed to influence mammalian microbiomes, although the influence of external factors, such as diet, can override these differences [90].

Related to general genotypic changes, host immunity can also shape microbial selection within a community. Innate immunity, present across plants and animals, is a host’s first line of defense and recognizes conserved patterns of microbes. Evolved to protect against pathogenic organisms that may harm the host, a host’s innate immunity has also evolved to select for and restrict microbes to specific niches [91]. In plants, root exudates with antimicrobial properties are used to reshape the microbiome and enhance resilience under stressful conditions [92]. Stimulation of the innate immune response in honey bees was found to support microbiome recovery after antibiotic treatment [93]. In vertebrate hosts, adaptive immunity provides an additional layer of defense through specialized immune cells and antibodies that target specific patterns, developed after pathogen exposure, effectively training the host to recognize and respond more efficiently during subsequent exposure. This response is slower than innate recognition, and can also be curated over time to more efficiently recognize and eliminate the threat. In mammals, specialized T cell populations are thought to restrict potentially harmful microbes as well as select for commensal microbiota, shaping its homeostasis trajectory [94]. For example, the adaptive host immune response has been demonstrated to drive recovery of a ‘healthy’ microbiota following fecal transplantation to treat *Clostridioides difficile* infection, which has been demonstrated to be an effective method for recovering colonization resistance against *C. difficile* [95].

Despite extensive evidence for the role of innate immunity and host genotype in shaping community structure, these factors are often excluded from ecological theories. In addition to individualized differences across a host’s range, the host immune response also evolves dynamically on a timeline independent of microbiome changes. As a result, incorporating these factors into ecological modeling is challenging, as they operate on separate trajectories from microbiome dynamics. Some advances in modeling approaches have tried to address this gap through incorporating host aspects. For example, Abbasi and Akçay applied a maximum microbial carrying capacity that a host is able to exhibit in their gLV model [96]. Others have applied predator–prey dynamics to explore resource-mediated competition between host-immune cells and pathogens [97], or eliminated immune system effects in their models by using organisms that lack an adaptive immune response to focus solely on microbiome changes [98]. Studies also demonstrate that the more complex the host, the more complex the community, once again suggesting that ecological theories may not be generalizable across host biomes [99]. Nevertheless, integrating immune and other host dynamics would greatly improve the use of ecological models to explain or predict composition.

### Incorporating Macro-ecological Theories to Host-associated Microbial Communities

Community ecological theories, while originally developed for larger-scale ecosystems, can offer valuable insights into microbial community dynamics when applied in appropriate contexts. The most effective applications of these theories are in controlled experimental systems, such as gnotobiotic models or more tightly regulated organ-on-a-chip platforms, where variables can be precisely manipulated to test ecological predictions but host influence is still partially accounted for [72, 100]. Additionally, longitudinal studies in healthy hosts offer a promising avenue for examining ecological patterns over time, particularly in stable environments where community assembly and succession can be systematically tracked [101]. Ideally, ecological theories based on model systems should be applied to studies in natural hosts to test the extent of ecological theory to describe natural phenomena and how well these models are preserved across different host microbiomes. Relatedly, the incorporation of ecological theory in biological studies, both experimental and observational, could strengthen our understanding of microbial community organization. This is especially true for host-associated microbiomes, which are known to exhibit dynamic changes over time, even across healthy hosts. Applying ecological frameworks to host-associated microbiomes could be especially useful when considering long-term evolutionary interactions between microbes and their hosts, providing a framework to assess stability, resilience, and adaptation [102]. By applying macro-scale ecological theories in these settings, we can build a stronger foundation for interpreting microbiome shifts in response to environmental change, ultimately improving our ability to predict and manipulate microbial community dynamics in both health and disease.

## Conclusion

The ability of classical ecological theories to understand microbiomes continues to improve as our mechanistic understanding and technological advances improve. Yet, the unique characteristics of microbial ecosystems—rapid growth rates, high genetic diversity, intimate host-microbe coevolution, and short evolutionary timescales—challenge traditional ecological frameworks. This is especially true for host-associated microbiomes, where theoretical explanations based from environmental microbiomes demonstrate their own unique restrictions. Looking forward, advancing our understanding of microbial community assembly and stability is particularly pressing in the face of escalating ecological disturbances, such as climate change, increased antibiotic usage, and emerging infectious diseases. As the stability of host-associated microbiomes is closely tied to host and ecosystem health, refining predictive models to include host-specific factors would enhance the frameworks we use to explain and predict microbiomes.

## Key References

- Leen B, Ward D, Gwen F, Sara V-S, Yhossef TR, Mireia V-C, Leen R, Daan J, Lore VE, Ioanna PM, Chenyan S, Kwe YC, Mark Z, Karoline F, Marc VR, Jeroen R, Jelle M. 2021. Successional Stages in Infant Gut Microbiota Maturation. mBio 12:e01857 - 21.
  - This study shows that infant gut microbiota maturation follows conserved transitions from oxygen-tolerant to strictly anaerobic microbes within the first year of life and find parallels between infant gut colonization and adult dysbiosis.
- Lynch CMK, Cowan CSM, Bastiaanssen TFS, Moloney GM, Theune N, van de Wouw M, Florensa Zanuy E, Ventura-Silva AP, Codagnone MG, Villalobos-Manríquez F, Segalla M, Koc F, Stanton C, Ross P, Dinan TG, Clarke G, Cryan JF. 2023. Critical windows of early-life microbiota disruption on behaviour, neuroimmune function, and neurodevelopment. Brain Behav Immun 108:309–327.
  - This study highlights that early-life antibiotic-induced microbiota disruption during critical developmental windows has lasting effects on the gut microbiome, immune cells, and neurophysiology.
- Fodelianakis S, Valenzuela-Cuevas A, Barozzi A, Daffonchio D. 2021. Direct quantification of ecological drift at the population level in synthetic bacterial communities. ISME J 15:55–66.
  - This study quantifies ecological drift in bacterial communities, revealing its role in growth variability, species loss, and β-diversity, particularly under high selection and low dispersal.
- Liou MJ, Miller BM, Litvak Y, Nguyen H, Natwick DE, Savage HP, Rixon JA, Mahan SP, Hiyoshi H, Rogers AWL, Velazquez EM, Butler BP, Collins SR, McSorley SJ, Harshey RM, Byndloss MX, Simon SI, Bäumler AJ. 2022. Host cells subdivide nutrient niches into discrete biogeographical microhabitats for gut microbes. Cell Host Microbe 30:836–847.e6.
  - This study shows that nutrient niches in the gut are biogeographically subdivided, with *Escherichia coli* and Salmonella enterica serovar Typhimurium occupying distinct nitrate-rich microhabitats, influencing strain abundance and colonization resistance.
- Shao C, Zhao W, Li N, Li Y, Zhang H, Li J, Xu Z, Wang J, Gao T. 2022. Gut Microbiome Succession in Chinese Mitten Crab Eriocheir sinensis During Seawater–Freshwater Migration. Front Microbiol 13.
  - This study found that salinity changes during migration from seawater to freshwater drive significant shifts in the gut microbiome of Chinese mitten crabs, affecting diversity, community composition, and metabolic pathways.
- Tarnecki AM, Miller C, Sherwood TA, Griffitt RJ, Schloesser RW, Wetzel DL. 2022. Dispersed Crude Oil Induces Dysbiosis in the Red Snapper Lutjanus campechanus External Microbiota.
  - This study found that chronic exposure to crude oil caused dysbiosis in juvenile red snapper's external microbiota increasing disease susceptibility.
- Lubin J-B, Green J, Maddux S, Denu L, Duranova T, Lanza M, Wynosky-Dolfi M, Flores JN, Grimes LP, Brodsky IE, Planet PJ, Silverman MA. 2023. Arresting microbiome development limits immune system maturation and resistance to infection in mice. Cell Host Microbe 31:554–570.e7
  - This study found that in mice, weaning-associated microbiome disruptions impair immune development and increase infection susceptibility.
- Kurkjian HM, Akbari MJ, Momeni B. 2021. The impact of interactions on invasion and colonization resistance in microbial communities. PLoS Comput Biol 17:e1008643-
  - This study proposes a mathematical model to predict microbiome invasion outcome that incorporates chemical-mediated interactions among residents and invaders.
- Brunner JD, Chia N. 2024. Metabolic model-based ecological modeling for probiotic design. Elife 13:e83690
  - This study examines probiotic colonization success by incorporating genome-scale metabolic modeling and Lotka-Volterra predictions.
- Xiong C, Zhu Y-G, Wang J-T, Singh B, Han L-L, Shen J-P, Li P-P, Wang G-B, Wu C-F, Ge A-H, Zhang L-M, He J-Z. 2021. Host selection shapes crop microbiome assembly and network complexity. New Phytologist 229:1091–1104
  - This study shows that crop microbiome assembly is driven primarily by plant compartment niche and host genotype.
- Henrick BM, Rodriguez L, Lakshmikanth T, Pou C, Henckel E, Arzoomand A, Olin A, Wang J, Mikes J, Tan Z, Chen Y, Ehrlich AM, Bernhardsson AK, Mugabo CH, Ambrosiani Y, Gustafsson A, Chew S, Brown HK, Prambs J, Bohlin K, Mitchell RD, Underwood MA, Smilowitz JT, German JB, Frese SA, Brodin P. 2021. Bifidobacteria-mediated immune system imprinting early in life. Cell 184:3884 - 3898.e11.
  - This study shows that early-life supplementation with *Bifidobacterium infantis* EVC001 promotes immunoregulation, reduces inflammation, and supports healthy immune-microbe interactions in breastfed infants.
- Jones EW, Carlson JM, Sivak DA, Ludington WB. 2022. Stochastic microbiome assembly depends on context. Proceedings of the National Academy of Sciences 119:e2115877119
  - This study shows that microbiome assembly in *Drosophila melanogaster* is shaped by stochastic, context-dependent bacterial interactions, contributing to variability even in controlled conditions.
- Lötstedt B, Stražar M, Xavier R, Regev A, Vickovic S. 2024. Spatial host–microbiome sequencing reveals niches in the mouse gut. Nat Biotechnol 42:1394–1403.
  - This study introduces a sequencing-based method that maps spatial host–microbiome interactions in tissues, revealing niche-specific gene programs and microbial geography in the gut.
- Coyte KZ, Rao C, Rakoff-Nahoum S, Foster KR. 2021. Ecological rules for the assembly of microbiome communities. PLoS Biol 19:1–19.
  - This study utilizes “assembly maps” to identify factors influencing microbiome assembly, highlighting the importance of both microbial and host interactions in shaping community success.
- Buttimer S, Moura-Campos D, Greenspan SE, Neely WJ, Ferrante L, Toledo LF, Becker CG. 2024. Skin microbiome disturbance linked to drought-associated amphibian disease. Ecol Lett 27:e14372.
  - This study found that drought-induced disruption of the skin microbiome in toads increased susceptibility a fungal pathogen, highlighting the impact of climate change on inter-kingdom dynamics.
- Weiss AS, Niedermeier LS, von Strempel A, Burrichter AG, Ring D, Meng C, Kleigrewe K, Lincetto C, Hübner J, Stecher B. 2023. Nutritional and host environments determine community ecology and keystone species in a synthetic gut bacterial community. Nat Commun 14:4780.
  - This study used strain dropouts in a synthetic bacterial community to show that keystone species influence microbiome dynamics in a context-dependent manner, varying by gut region and environment.
- Jurburg SD, Blowes SA, Shade A, Eisenhauer N, Chase JM. 2024. Synthesis of recovery patterns in microbial communities across environments. Microbiome 12:79.
  - This meta-analysis found that microbiome recovery after disturbance is environment-specific, with mammalian microbiomes regaining richness but not composition and aquatic microbiomes diverging further over time.
- Sun Q, Vega NM, Cervantes B, Mancuso CP, Mao N, Taylor MN, Collins JJ, Khalil AS, Gore J, Lu TK. 2022. Enhancing nutritional niche and host defenses by modifying the gut microbiome. Mol Syst Biol 18:e9933.
  - This study engineered microbiomes in *Caenorhabditis elegans* demonstrating the potential to enhance host metabolic and protective functions through microbiome modifications.

## Data Availability

No datasets were generated or analysed during the current study.
